# Late presentation of a mucinous ovarian adenocarcinoma which was initially diagnosed as a primary pancreatic carcinoma: a case report and review of the literature

**DOI:** 10.1186/1752-1947-4-90

**Published:** 2010-03-18

**Authors:** Dorothy A Sparks, Daniel M Chase, Mark Forsyth, Gregg Bogen, Jon Arnott

**Affiliations:** 1 Department of Surgery, Northside Medical Center, Gypsy Lane, Youngstown, Ohio, 44505, USA; 2 Department of Pathology, Northside Medical Center, Gypsy Lane, Youngstown, Ohio, 44505, USA; 3 Department of Internal Medicine, Northside Medical Center, Gypsy Lane, Youngstown, Ohio, 44505, USA

## Abstract

**Introduction:**

Adenocarcinoma of the ovary is an aggressive neoplasm which often metastasizes to the lung or liver. Metastases rarely occur to the pancreas, but a tissue diagnosis is required to confirm this event. Although most tumors of the pancreas are primary pancreatic neoplasms, metastatic lesions have been reported most commonly as arising from renal cell carcinoma.

**Case presentation:**

We report the case of a 51-year-old Caucasian woman with ovarian mucinous adenocarcinoma with metastasis to the head of the pancreas that was originally misdiagnosed as a pancreatic primary tumor.

**Conclusion:**

Mucinous ovarian adenocarcinomas rarely metastasize to the pancreas. New pancreatic lesions should be investigated through tissue biopsy and tumor markers, while keeping an open-minded differential diagnosis to avoid a misdiagnosis or a delay in treatment.

## Introduction

Although most malignant tumors of the pancreas are primary pancreatic neoplasms, metastatic lesions have been reported most commonly as arising from renal cell carcinoma. Here we present a case of mucinous adenocarcinoma of the ovary that metastasized to the pancreas. The tumor was first diagnosed as a primary pancreatic tumor. Ovarian adenocarcinoma can have distant metastases, but these are most often to the liver or lung. Metastasis to the pancreas is quite rare, and a delay in its diagnosis may occur if the pancreatic tumor is not identified as a metastatic disease.

## Case presentation

A 51-year-old Caucasian woman complained of fatigue, epigastric discomfort, a left neck mass, and a 10-pound weight loss over the previous six months. Her physical examination revealed supraclavicular lymphadenopathy.

A cervical lymph biopsy revealed moderately well-differentiated adenocarcinoma, possibly of pancreatic origin. A metastatic workup including positron emission tomography (PET) scan, computed tomography (CT), bone scan, and breast and pelvic ultrasounds was done. Significant cervical, retrosternal and retroperitoneal lymphadenopathy were seen. A 3.5 × 5-cm pancreatic head mass which blended into the porta hepatis was also noted. The mass encased the left gastric artery and involved the portal vein margins. Multiple liver lesions were also seen. Except for a fibroid uterus, her pelvic CT and ultrasound were unremarkable.

A percutaneous liver biopsy of our patient revealed a moderately well-differentiated adenocarcinoma consistent with pancreatic origin. Her CA-19-9 level was 171.5.

Our patient was then entered into a trial for advanced pancreatic adenocarcinoma using the tyrosine kinase inhibitor Dasatinib. Her response to the trial, however, was poor and a CT scan two months later showed no reduction in her tumor burden. She was removed from the Dasatinib trial and was started on Gemzar (gemcitabine) and Tarceva (erlotinib).

Over the next seven months, our patient developed a moderate response to chemotherapy. However, she developed abdominal fullness and shortness of breath. In just a four-month interval between scans, her CT revealed a new 19 × 18 × 9 cm pelvic mass, ascites, and a large right-sided pleural effusion. A CT-guided biopsy revealed poorly differentiated adenocarcinoma. Metastatic malignant cells were also found in her pleural fluid.

Because our patient experienced significant discomfort due to the effects of her pelvic mass, a palliative resection was performed. She had a transient response to chemotherapy, but her disease continued to progress with worsening ascites and pleural effusion.

**Figure 1:**
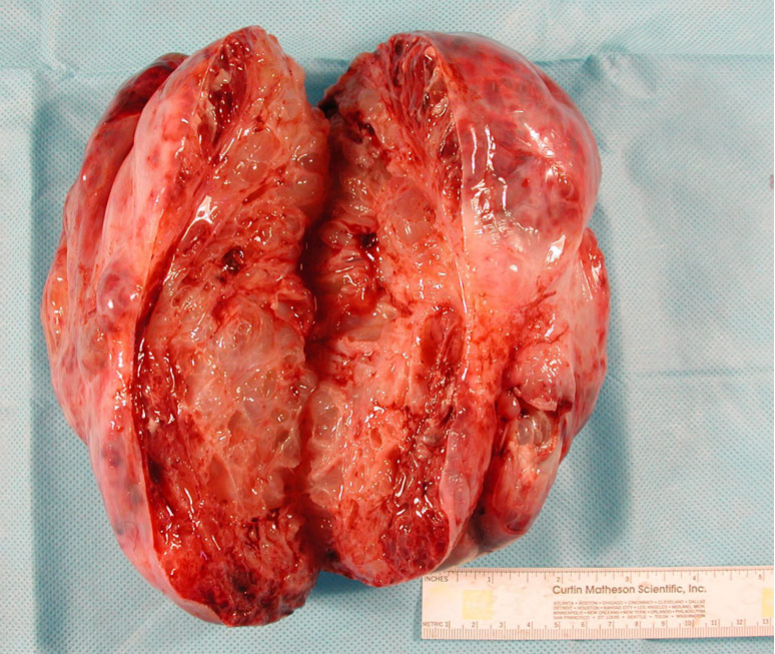
**Gross specimen with clearly defined cystic and mucinous component (40× magnification)**.

**Figure 2:**
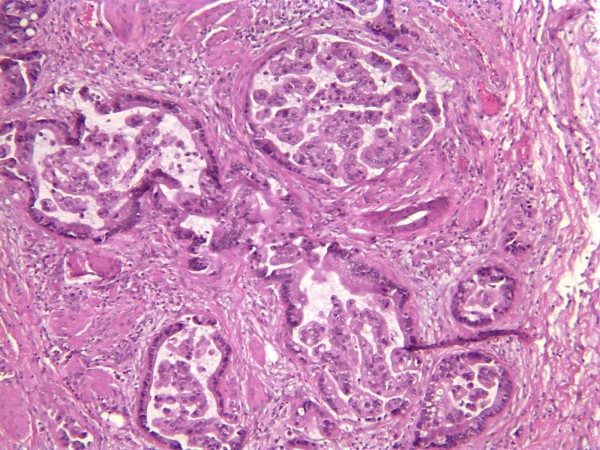
**Hematoxylin and eosin slide of the ovarian mucinous carcinoma demonstrating lymphatic invasion (40× magnification)**.

Pathology revealed that her pelvic mass measured 19.0 × 18.0 × 9.0 cm. Sectioning revealed a multi-loculated cystic mass involving her entire ovary (Figure 1). No normal ovarian tissue was identified on gross examination. On histology the tumor was found to consist of a complex formation of dilated cystic glands filled with mucin. The mucinous cysts were lined by a layer of columnar mucinous cells with pale to clear cytoplasm and a small, bland, basally situated oval nuclei. This finding was consistent with a borderline mucinous tumor. There were areas of invasion of her fallopian tube and lymphatics (Figure 2). The tumor was also found surrounding her residual ovarian stroma at the periphery.

**Figure 3:**
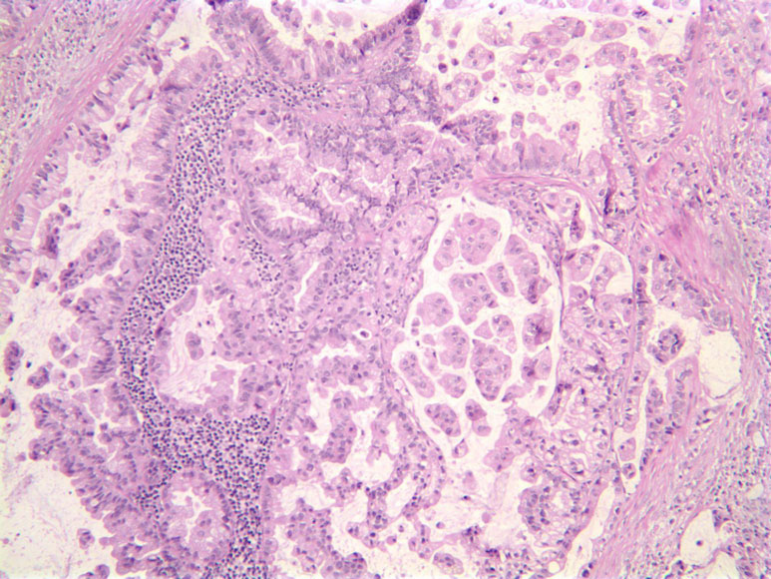
**Hematoxylin and eosin slide of our patient's lymph node and metastasis (40× magnification)**.

In view of the presence of metastatic adenocarcinoma to the liver and a cervical lymph node (Figure 3), as well as the pancreatic mass found on CT, we initially identified the primary tumor as pancreatic. Unfortunately, a fine needle aspiration of the pancreas was not diagnostic.

When her ovarian mass stained positive for CK7 but negative for CK20 and estrogen receptor, we repeated the stains on her liver and lymph node biospies, which proved to be a match to the ovarian tumor.

The histologic and immunohistochemical findings of our patient's ovarian mass are consistent with the results of her lymph node and liver biopsies. We concluded that the mass was most likely from the ovary and not the pancreas, with the pancreatic mass representing another metastasis of her ovarian adenocarcinoma.

## Discussion

Mucinous ovarian adenocarcinomas are uncommon and account for only 10% to 15% of reported cases of ovarian neoplasms [1]. Most mucinous ovarian tumors are considered borderline with low malignant potential [2]. Tumors that metastasize are often poorly differentiated and of the low-grade type [1].

Often characterized by multiseptated cystic lesions of the adenexa, mucinous adenocarcinomas are filled with a gelatinous material that may freely rupture into the peritoneal cavity, thus causing a dissemination called "pseudomyxoma peritoni" [2]. Pseudomyxoma peritoni can arise from any other mucinous-type adenocarcinomas, including those of the appendix, breast, prostate, and colon [1]. Ovarian neoplasms may also metastasize to the lung or the liver [3] through lymphatic spread via the deep inguinal nodal basin.

Carcinoma metastatic to the pancreas is uncommon and arises by direct extension from retroperitoneal or mesenteric lymph nodes or from isolated metastases to the pancreatic parenchyma [4]. The most common primary origin of solitary pancreatic metastases is renal cell carcinoma, [5,6] but other sources include the lung and the colon [7]. Pancreatic metastasis from a gynecologic primary is rare [4]. However, the incidence in advanced ovarian tumors may be higher than had been previously considered. In fact one autopsy study showed pancreatic metastases in 21% of patients with ovarian cancer [8]. The pancreatic head remains the most common site of metastasis [5].

Due to the low incidence of pancreatic metastasis, most masses of the pancreas are assumed to be primary pancreatic neoplasms. However, a tissue biopsy is required to truly differentiate between primary and secondary tumors [1]. A delay in diagnosis can occur when this assumption is not verified by biopsy, as in the case of a 72-year-old woman reported by Schumacher [9]. Her pancreatic mass was not recognized as ovarian until 10 months after its initial discovery. The lack of an adenexal mass to initially raise suspicion of an ovarian primary tumor may also contribute to a delay in diagnosis, as in our case.

Differentiating primary from secondary pancreatic tumors is important in directing a patient's therapy, both in terms of chemotherapy and surgery. Whenever possible, the resection of pancreatic metastasis can be a reasonably safe palliative procedure [4]. Distal pancreatic resection of metastatic ovarian cancers has been shown to be beneficial, even if found incidentally during a debulking procedure [10].

## Conclusion

Although most lesions of the pancreas are primary pancreatic neoplasms, a tissue biopsy should be obtained whenever possible to differentiate between primary and secondary tumors. Metastases of ovarian mucinous adenocarcinomas to the pancreas are rare, but have been reported in the literature. Confirmatory tissue biopsies, tumor markers, and being mindful of the possibility of metastatic disease can avoid misdiagnosis and delay in treatment for newly discovered pancreatic masses.

## Consent

Written informed consent was obtained from the patient for publication of this case report and any accompanying images. A copy of the written consent is available for review by the Editor-in-Chief of this journal.

## Competing interests

The authors declare that they have no competing interests.

## Authors' contributions

DS researched the case and was a major contributor in writing the manuscript, particularly the case discussion. DC performed research, contributed in writing the case report, and edited the manuscript for its final version. MF performed the histological examination described in the case report and was a major contributor in writing the manuscript. GB was our patient's attending surgeon and provided information on our patient and contributed to the writing of the manuscript. JA was the primary care provider involved in the case, and similarly provided patient information and contributed in writing the manuscript. All authors read and approved the final manuscript.

## References

[B1] Kumar V Robbins and Cotran Pathologic Basis of Disease 2004 7 Philadelphia: Saunders

[B2] Berek JS Hacker NF Practical Gynecologic Oncology 2000 4 Philadelphia: Lippincott Williams and Wilkins

[B3] Yilmaz Z Bese T Demirkiran F Ilvan S Sanioglu C Arvas M Kosebay D Skin metastasis in ovarian carcinoma In J Gynecol Cancer 2006 16 414 418 10.1111/j.1525-1438.2006.00486.x 16515636

[B4] Pingpank JF Hoffman JP Sigurdson ER Ross E Sasson AR Eisenberg BL Pancreatic resection for locally advanced primary and metastatic nonpancreatic neoplasms Am Surg 2002 68 337 341 11952243

[B5] Silva RG Dahmoush L Gerke H Pancreatic metastasis of an ovarian malignant mixed mullerian tumor identified by EUS-guided fine needle aspiration and trucut needle biopsy J Pancreas 2006 7 66 69 16407622

[B6] Robbins EG Franceschi D Barkin JS Solitary metastatic tumors to the pancreas: a case report and review of the literature Am J Gastroenterol 1996 91 2414 2417 8931428

[B7] Roland CF van Heerden JA Non-pancreatic primary tumors with metastasis to the pancreas Surg Gynecol Obstet 1989 168 345 347 2928909

[B8] Dvoretsky PM Richards KA Angel C Rabinowitz L Stoler MH Beecham JB Bonfiglio TA Distribution of disease at autopsy in 100 women with ovarian cancer Hum Pathol 1988 19 57 63 10.1016/S0046-8177(88)80316-2 3335391

[B9] Schumacher A Delayed diagnosis of ovarian cancer with metastasis to the pancreas Zentrabl Gynakol 1993 115 568 569 8147172

[B10] Yildirim Y Sanci M The feasibility and morbidity of distal pancreatectomy in extensive cytoreductive surgery for advanced epithelial ovarian cancer Arch Gynecol Obstet 2005 272 31 34 10.1007/s00404-004-0657-3 15480722

